# Extended endoscopic endonasal transplanum transtuberculum approach for retrochiasmatic type II transinfundibular craniopharyngioma: operative pearls and nuances of the technique

**DOI:** 10.3171/2026.7.FOCVID26130

**Published:** 2026-10-01

**Authors:** James K. Liu, Jean Anderson Eloy

**Affiliations:** 1 Department of Neurosurgery, Cooperman Barnabas Medical Center, RWJ Barnabas Health, Livingston;; 2 Skull Base Institute of New Jersey, Neurosurgeons of New Jersey, Livingston;; 3 Department of Otolaryngology and Facial Plastic Surgery, Cooperman Barnabas Medical Center, RWJ Barnabas Health, Livingston; and; 4 Department of Otolaryngology–Head and Neck Surgery, Rutgers New Jersey Medical School, Newark, New Jersey

**Keywords:** craniopharyngioma, retrochiasmatic, endoscopic skull base surgery, endoscopic endonasal approach, transsphenoidal, operative video

## Abstract

Type II transinfundibular retrochiasmatic craniopharyngiomas present significant surgical challenges because they infiltrate and expand the pituitary stalk, usually making functional preservation unfeasible. The authors present an operative video demonstrating an endoscopic endonasal transplanum transtuberculum resection of a complex transinfundibular craniopharyngioma in a 62-year-old female who presented with progressive vision loss. Key technical nuances included mobilizing the arachnoid veil to protect optic chiasm perforators, creating a transglandular transinfundibular window to widen the infrachiasmatic corridor, dissecting the tumor-hypothalamic interface, and performing vascularized multilayered reconstruction. Gross-total resection was achieved without cerebrospinal fluid leakage. The patient’s vision significantly improved, and panhypopituitarism was effectively managed with hormone replacements.

The video can be found here: https://stream.cadmore.media/r10.3171/2026.7.FOCVID26130

## Transcript

This is Dr. James Liu, and I will be demonstrating the extended endoscopic endonasal transplanum transtuberculum approach for resection of a retrochiasmatic transinfundibular craniopharyngioma.

### 0:34 Clinical Presentation and Imaging.

The patient is a 62-year-old female who presented with progressive visual loss and increased thirst and water intake. Visual field exam showed bitemporal hemianopsia. Neuro exam was otherwise normal. Preoperative endocrine workup showed normal pituitary function. Preoperative MRI demonstrated a 2.2-cm heterogeneously enhancing suprasellar retrochiasmatic tumor most consistent with a craniopharyngioma.

### 1:02 Surgical Strategy and Planning.

The tumor was removed using an extended endoscopic endonasal transplanum transtuberculum approach. Based on the preoperative imaging, we suspected that this was possibly a type II transinfundibular craniopharyngioma where the tumor grows and expands the stalk.^1,2^ These findings were confirmed at surgery, and we used a transglandular transinfundibular surgical corridor. The operative pearls listed here will be highlighted in the video.

### 1:30 Patient Positioning.

After placing a lumbar drain, the patient is positioned supine. The nose and nares are prepped with Betadine solution, and the right thigh is prepped for a fascia lata graft harvest. Intraoperative frameless electromagnetic neuronavigation and neurophysiological monitoring with visual evoked and somatosensory evoked potentials are used throughout the case.

### 1:50 Endoscopic Endonasal Exposure.

Using a 30° angled endoscope, the initial endonasal exposure begins with lateralizing the right middle turbinate and entering the right sphenoid ostia to widen the sphenoid sinus. A posterior ethmoidectomy is also performed to widen the corridor. We then lateralize the left middle turbinate and perform a left sphenoidotomy and posterior ethmoidectomy. The superior olfactory strips are elevated bilaterally to optimize preservation of olfactory function. We prefer to harvest the nasoseptal flap at the time of closure to tailor the flap dimensions to the specific defect. As such, we use a rescue flap technique to preserve the vascular pedicle until the reconstruction phase.

### 2:43 Transplanum Transtuberculum Exposure.

We then proceed to drill off the bony sellar floor, the tuberculum sellae, and the planum sphenoidale using eggshelling technique with a diamond burr. Care is taken to avoid injury to the paraclinoid segment of the internal carotid arteries.

### 3:09 Dural Opening and Exposure of Suprasellar Tumor.

The sellar dura is coagulated and opened sharply with an arachnoid knife and angled scissors. The superior intercavernous sinus is coagulated with an endoscopic bipolar, and the transplanum dura is opened sharply. The intercavernous sinus is divided sharply to access the tumor in the suprasellar cistern. There is a veil of arachnoid over the tumor capsule, which is opened and mobilized laterally to preserve the integrity of the superior hypophyseal perforators supplying the undersurface of the chiasm. This is important to preserve visual function. Intraoperative inspection confirms our suspicion that the tumor is a type II transinfundibular craniopharyngioma. The tumor appears to be originating from the tuber cinereum, which represents the most cephalad, intrinsic variant of type II transinfundibular tumors. They grow predominantly along the tuberoinfundibular axis and frequently expand upward into the third ventricle. As such, pituitary stalk preservation in these cases is exceptionally difficult and often unfeasible. Alternate strategies to consider are pituitary gland transposition to access the tumor or settling for subtotal resection. However, panhypopituitarism can occur even with an anatomically preserved stalk with residual tumor. Because we determined that the stalk was not preservable, our strategy was to widen the corridor by creating a transglandular transinfundibular window.

### 4:57 Tumor Removal.

Intratumoral debulking is initially performed, and a frozen section came back as craniopharyngioma. Under direct endoscopic visualization, extracapsular dissection is then performed. We initially develop a plane along the superior margin and bring the tumor down inferiorly with an up-angled loop curette. One of the major advantages of the endonasal approach is the ability to have direct visualization of the undersurface of the optic chiasm and hypothalamus so that safe microdissection can be performed to carefully separate the tumor capsule from the hypothalamic surface. Further piecemeal debulking is performed with curettes and pituitary rongeurs as the tumor is somewhat soft and friable. With gentle traction on the tumor capsule, we use suction dissection to sweep the tumor off of the right hypothalamic interface. We then elevate the tumor from the membrane of Liliequist and dissect along the inferior surface of the tumor to identify the mamillary bodies. Once the tumor adhesions are freed up from the hypothalamus, we can take advantage of the free edge of the tumor and use an angled ring curette to hook around the tumor and deliver it from back to front. It is important to avoid blind dissection and do this under direct endoscopic visualization. We continue to deliver the tumor in a piecemeal fashion using an angled ring curette and pituitary rongeurs. The small perforating vessels to the tumor are carefully coagulated and divided sharply to avoid avulsing them from its origin. A gross-total resection is achieved, and final inspection with the endoscope reveals a spectacular view of the retrochiasmatic anatomy including the membrane of Liliequist, mamillary bodies, cerebral acqueduct, hypothalamus, and third ventricle.

### 7:31 Multilayered Skull Base Reconstruction (Closure).

We then harvest a left-sided vascularized pedicled nasoseptal flap for reconstruction. It is important to take the flap along the floor of the nose to maximize the surface area for coverage of the extended transplanum skull base defect. Multilayered reconstruction is performed, initially with a DuraGen inlay graft tucked underneath the dural edges. This is followed by two layers of autologous fascia lata onlay grafts placed extradurally that is secured with Surgicel. It is important to leave the surrounding bone exposed for the nasoseptal flap to make contact with to optimize flap adherence. The vascularized pedicled nasoseptal flap is then rotated and carefully positioned to ensure maximal contact to the bony skull base for complete coverage of the skull base repair. We bolster the reconstruction with Surgicel, followed by gentamicin-soaked Gelfoam pledgets, and buttress this with a Merocel pack wedged into the sphenoid sinus.

### 8:45 Postoperative Imaging and Clinical Course.

Postoperative MRI demonstrated no evidence of residual tumor. The optic chiasm was nicely decompressed. Postoperatively, the patient was neurologically intact with improved vision and visual fields. As expected, she had panhypopituitarism and was placed on hormone replacement therapy. Lumbar drainage was continued for 48 hours. There was no CSF leakage, and the patient was discharged on postoperative day 6. Final pathology revealed adamantinomatous craniopharyngioma.

### 9:19 Conclusions.

In conclusion, the endoscopic transplanum transtuberculum approach is a safe and effective approach for retrochiasmatic craniopharyngiomas.^3–6^ The pituitary stalk is usually not preservable in transinfundibular craniopharyngiomas when attempted maximal safe gross-total resection is the goal. Performing a transglandular transinfundibular window is an efficient alternative to pituitary transposition to widen the infrachiasmatic corridor in these scenarios.

## Disclosures

The authors report no conflict of interest concerning the materials or methods used in this study or the findings specified in this publication.

## Author Contributions

Primary surgeon: Liu. Assistant surgeon: Eloy. Editing and drafting the video and abstract: both authors. Critically revising the work: both authors. Reviewed submitted version of the work: both authors. Approved the final version of the work on behalf of both authors: Liu. Supervision: both authors.

## Supplemental Information

### Patient Informed Consent

The necessary patient informed consent was obtained in this study.
